# Predictive scores for identifying patients with type 2 diabetes mellitus at risk of acute myocardial infarction and sudden cardiac death

**DOI:** 10.1002/edm2.240

**Published:** 2021-02-19

**Authors:** Sharen Lee, Jiandong Zhou, Cosmos Liutao Guo, Wing Tak Wong, Tong Liu, Ian Chi Kei Wong, Kamalan Jeevaratnam, Qingpeng Zhang, Gary Tse

**Affiliations:** ^1^ Cardiovascular Analytics Group Laboratory of Cardiovascular Physiology Hong Kong China; ^2^ School of Data Science City University of Hong Kong Hong Kong Hong Kong China; ^3^ Li Ka Shing Institute of Health Sciences Chinese University of Hong Kong Hong Kong China; ^4^ School of Life Sciences Chinese University of Hong Kong Hong Kong China; ^5^ Tianjin Key Laboratory of Ionic‐Molecular Function of Cardiovascular disease Department of Cardiology Tianjin Institute of Cardiology Second Hospital of Tianjin Medical University Tianjin China; ^6^ Department of Pharmacology and Pharmacy University of Hong Kong Pokfulam Hong Kong China; ^7^ Medicines Optimisation Research and Education (CMORE UCL School of Pharmacy London UK; ^8^ Faculty of Health and Medical Sciences University of Surrey Guildford UK

## Abstract

**Introduction:**

The present study evaluated the application of incorporating non‐linear J/U‐shaped relationships between mean HbA1c and cholesterol levels into risk scores for predicting acute myocardial infarction (AMI) and non‐AMI‐related sudden cardiac death (SCD) respectively, amongst patients with type 2 diabetes mellitus.

**Methods:**

This was a territory‐wide cohort study of patients with type 2 diabetes mellitus above the age 40 and free from prior AMI and SCD, with or without prescriptions of anti‐diabetic agents between January 1st, 2009 to December 31st, 2009 at government‐funded hospitals and clinics in Hong Kong. Patients recruited were followed up until 31 December 2019 or their date of death. Risk scores were developed for predicting incident AMI and non‐AMI‐related SCD. The performance of conditional inference survival forest (CISF) model compared to that of random survival forests (RSF) model and multivariate Cox model.

**Results:**

This study included 261 308 patients (age = 66.0 ± 11.8 years old, male = 47.6%, follow‐up duration = 3552 ± 1201 days, diabetes duration = 4.77 ± 2.29 years). Mean HbA1c and low high‐density lipoprotein‐cholesterol (HDL‐C) were significant predictors of AMI on multivariate Cox regression. Mean HbA1c was linearly associated with AMI, whilst HDL‐C was inversely associated with AMI. Mean HbA1c and total cholesterol were significant multivariate predictors with a J‐shaped relationship with non‐AMI‐related SCD. The AMI and SCD risk scores had an area under the curve (AUC) of 0.666 (95% confidence interval (CI) = [0.662, 0.669]) and 0.677 (95% CI = [0.673, 0.682]), respectively. CISF significantly improves prediction performance of both outcomes compared to RSF and multivariate Cox models.

**Conclusion:**

A holistic combination of demographic, clinical and laboratory indices can be used for the risk stratification of patients with type 2 diabetes mellitus for AMI and SCD.

## Graphical abstract

We evaluated the application of incorporating non‐linear J/U‐shaped relationships between both mean HbA1c and cholesterol levels into risk scores for predicting for acute myocardial infarction (AMI) and non‐AMI‐related sudden cardiac death (SCD), respectively. The performance of conditional inference survival forest (CISF) model compared to that of random survival forests (RSF) model and multivariate Cox model.

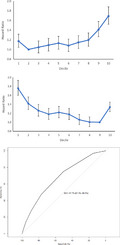

## 1 INTRODUCTION

Type 2 diabetes mellitus is an increasingly prevalent disease burden across the globe due to ageing and lifestyle westernization, with numbers projected to increase by up to 439 million by 2030.^1^ Diabetes mellitus is burdensome to the healthcare system for its chronic course and a multitude of possibly debilitating and lethal complications across different organ systems. Acute myocardial infarction (AMI) and sudden cardiac death (SCD) are major cardiovascular adverse outcomes in patients with type 2 diabetic mellitus.^2^, ^3^

Given the potentially lethal and debilitating nature of such cardiovascular adverse outcomes, many risk scores have been developed in hopes of identifying high‐risk patients for early intervention and close monitoring. For example, the UKPDS Risk Engine is a type 2 diabetes mellitus‐specific risk score based on the United Kingdom Prospective Diabetes Study (UKPDS) for ischaemic heart disease.^4^ The Reynolds Risk Score was developed to assess female cardiovascular risk, and the China‐PAR project was devised to target the Chinese population specifically.^5^, ^6^ However, typically these risk scores involving HbA1c and lipid level predicted for composite outcomes of major cardiovascular adverse outcomes or cardiovascular mortality, which did not account for the difference in pathogenesis and prognosis between acute coronary syndrome and lethal ventricular arrhythmias. Furthermore, recent studies reported that HbA1c and lipid levels, which were often accounted for in these risk scores, have J/U‐shaped relationships with adverse outcomes.^7^, ^8^, ^9^, ^10^ Therefore, updated risk scores that incorporate these new findings for predictions of specific cardiovascular adverse outcomes were warranted for personalized management.

The present study evaluated the application of incorporating non‐linear J/U‐shaped relationships between both mean HbA1c and cholesterol levels into risk scores for predicting AMI and non‐AMI‐related SCD respectively, amongst type 2 diabetes mellitus patients. A conditional inference survival forests (CISF) model was used for time‐to‐event survival data analysis in predicting AMI and non‐AMI SCD.^11^, ^12^

## 2 METHODS

### 2.1 Study design

The present study has been approved by The Joint Chinese University of Hong Kong—New Territories East Cluster Clinical Research Ethics Committee. Patients fulfilling all of the following inclusion criteria were recruited: 1) above the age 40; 2) had documented diagnosis of type 2 diabetes mellitus under the International Classification of Disease, Ninth Edition (ICD‐9) coding system, or prescribed anti‐diabetic agents between 1 January 2009 to 31 December 2009 by any of the Hong Kong Hospital Authority‐managed public hospitals or outpatient clinics; 3) without prior history of AMI and SCD episodes. The data were collected from the Clinical Data Analysis and Reporting System (CDARS), an electronic medical database that integrates patient data for shared comprehensive healthcare records to be across the public hospitals and clinics. The system has been used for cohort studies by both the present research team and other teams in the past.^13^, ^14^, ^15^, ^16^

### 2.2 Data extraction

The primary outcome of the present study, the time to the initial AMI and non‐AMI‐related SCD episode, is defined as days from 1st January 2009 to the date of initial AMI/ non‐AMI‐related SCD or the end of the follow‐up period (31 December 2019). A SCD episode is defined as an episode of sustained ventricular tachycardia, ventricular fibrillation or non‐specific cardiac arrest. This includes episodes that were aborted (sudden cardiac arrest) and episodes that resulted in death. SCD episodes with AMI within a week before or after the SCD episode were considered AMI‐related and thus excluded. The number of AMI and non‐AMI‐related SCD episodes during the follow‐up period was extracted as well. Other clinical characteristics, including demographic details (age and sex), diabetes duration, pre‐existing comorbidities, anti‐diabetic agents, and cardiovascular agents prescribed, and all‐cause mortality, were also extracted. The onset of diabetes is determined by fulfilment of the following criteria, whichever is the earliest: 1) earliest record of type 2 diabetes mellitus‐related ICD‐9 codes; 2) earliest record of HbA1c >6.5%; 3) earliest record of fasting blood glucose (FBG) >7 mmol/L. The duration of diabetes is defined as the onset of diabetes until 31 December 2009. Similarly, follow‐up duration was defined as from 1 January 2009 to 31 December 2019 or the date of death.

The following pre‐existing comorbidities were identified using ICD‐9 codes (Table S1): 1) renal, ophthalmological and neurological diabetic comorbidities; 2) heart failure (HF); 3) atrial fibrillation (AF); 4) hypertension; 5) peripheral vascular disease (PVD); 6) ischaemic stroke; 7) osteoporosis; 8) chronic obstructive pulmonary disease (COPD); 9) ischaemic heart disease (IHD). The classes of anti‐diabetic agents extracted were as follows: 1) biguanide; 2) sulphonylurea; 3) insulin; 4) dipeptidyl peptidase‐4 inhibitor (DPP4I); 5) glucagon‐like peptide‐1 agonist (GLPA); 6) meglitinide; 7) alpha‐glucosidase inhibitor; 8) thiazolidinedione. Antihypertensives (angiotensinogen‐converting enzyme inhibitor (ACEI)/ angiotensin receptor blocker (ARB), beta‐adrenergic receptor blocker, calcium channel blocker (CCB), diuretics) and lipid‐lowering agents were also extracted.

Baseline laboratory data from complete blood count (lymphocyte, neutrophil count and haemoglobin level), liver function test (alanine aminotransferase (ALT), alkaline phosphatase (ALP), albumin and total protein), renal function test (creatinine, sodium, potassium, urea), lipid (high‐density lipoprotein‐cholesterol (HDL‐C), low‐density lipoprotein‐cholesterol (LDL‐C), total cholesterol, triglyceride) and glycaemic profile (FBG, HbA1c) between 1 January 2008 to 31 December 2008 were obtained. Baseline anaemia was defined as haemoglobin count <13 g/dL amongst male, and <12 g/dL amongst female. Mean HbA1c and FBG from 1 January 2004 to 31 December 2008 were also calculated.

### 2.3 Statistical analysis

The annualized rate and mean event frequency were calculated for the primary outcomes. The annualized rate was calculated by dividing the total number of episodes across the cohort by the number of patient‐years follow‐up. The mean event annual frequency was calculated by averaging the individual mean number of episodes per year throughout follow‐up amongst those who experienced the event. Univariate Cox regression was used to identify predictors for incident episodes of both AMI and non‐AMI‐related SCD. Patients with AMI prior to non‐AMI‐related SCD were excluded for the SCD analysis. Hazard ratio (HR), 95% confidence interval (CI) and *P* value were reported for the Cox regression. Univariate predictors with *P *< 0.10 were entered into a multivariate model. Significant predictors were then selected into predictive scores. The multivariate Cox regression was then repeated with only the significant predictors to obtain the HR for adjustments for the score. For variables with HR between 0.67 and 1.5, a score of 1 was assigned, otherwise a score of 2 was assigned.

To examine the potential incorporation of the J/U‐shaped relationship reported between glycaemic/cholesterol profile and cardiovascular adverse events, the deciles of these parameters that were included in the score were obtained and used to derive the HR predicting for AMI and non‐AMI‐related SCD respectively through univariate Cox regression. Then, the decile with the minimal HR, excluding the first and last decile, was selected as the reference decile and compared against the remaining deciles. Univariate Cox regression was then repeated, and the derived HR was plotted. Parameters that displayed a J/U‐shaped relationship with the selected outcome would have had the score adjusted for, with the minimum and maximum cut‐offs derived deciles that had a statistically insignificant difference in HR with the reference decile. The cut‐off for other continuous variables included in the score was derived by maximizing the sensitivity and specificity. To evaluate the scores, a receiver operating characteristic (ROC) curve was then generated for the scores, and the area under the curve (AUC) was calculated. Statistical significance was defined as *P* < 0.05. The statistical analysis was performed using RStudio software (Version: 1.1.456).

## 3 RESULTS

### 3.1 Baseline characteristics

This study included 261308 patients (age = 66.0 ± 11.8 years old, male = 47.6%, follow‐up duration = 3552 ± 1201 days, diabetes duration = 4.77 ± 2.29 years). The categorical and continuous baseline demographic, clinical and laboratory features are presented in Tables 1 and 2, respectively. The mean HbA1c level was 7.67 ± 1.17%, with anaemia present in 14.3% of the cohort at baseline. On follow‐up, 33.3% (n = 86 908) of the patients died. The five most prevalent comorbidities in decreasing order are hypertension (23.1%), IHD (7.7%), HF (3.5%), ischaemic stroke (3.3%), and AF (2.8%). On average, patients had 0.44 ± 0.80 of the extracted comorbidities. In terms of drug use, most patients were on monotherapy or combination therapy of biguanide (69.0%), sulphonylurea (64.0%) and insulin (10.4%), on average on 1.45 ± 0.80 anti‐diabetic agents. ACEI/ARB (19.0%) was the most common class of antihypertensive prescribed, followed by CCB (17.4%) and beta‐adrenergic receptor blocker (14.6%). Lipid‐lowering agents were prescribed in 10.6% of the patients. On average, patients from the present cohort were on 1.58 ± 1.27 cardiovascular medications.

**TABLE 1:** Baseline characteristics for categorical variables in the present cohort

Characteristics	Number (%)		
	Total cohort (n = 261 308)	Acute myocardial infarction (n = 20 419)	Sudden cardiac death (n = 12 282)
Male	124 495 (47.6)	10 221 (50.1)	6454 (52.5)
Mortality	86 908 (33.3)	14374 (70.4)	12 096 (98.5)
Acute myocardial infarction (AMI)	20 419 (7.81)	—	—
Sudden cardiac death (SCD)	12 282 (4.74)	—	—
Baseline anaemia	37 286 (14.3)	5048 (24.7)	3470 (28.3)
Anti‐diabetic agent			
Biguanide	180 232 (69.0)	13 797 (67.6)	7776 (63.3)
Sulphonylurea	167 174 (64.0)	14 421 (70.6)	8684 (70.7)
Insulin	27 269 (10.4)	3620 (17.7)	2115 (17.2)
Meglitinide	25 (0.010)	3 (0.015)	3 (0.024)
Dipeptidyl Peptidase−4 Inhibitor	316 (0.121)	22 (0.108)	10 (0.081)
Thiazolidinedione	3741 (1.43)	335 (1.64)	162 (1.32)
Glucagon‐like Peptide−1 Agonist	15 (0.006)	0 (0)	0 (0)
Acarbose	3119 (1.19)	404 (1.98)	218 (1.77)
Cardiovascular drugs			
Angiotensinogen‐converting enzyme inhibitor (ACEI)/ angiotensin receptor blocker (ARB)	49 712 (19.0)	5769 (28.3)	3363 (27.4)
Beta‐adrenergic receptor blocker	38 144 (14.6)	4577 (22.4)	2524 (20.6)
Calcium channel blocker	45 542 (17.4)	5604 (27.4)	3265 (26.6)
Diuretic	24 204 (9.26)	3209 (15.7)	2079 (16.9)
Lipid‐lowering agent	27 828 (10.6)	3797 (18.6)	1932 (15.7)
Comorbidities			
Renal diabetic complication	3049 (1.17)	563 (2.76)	382 (3.11)
Peripheral vascular disease (PVD)	299 (0.114)	78 (0.382)	33 (0.269)
Ophthalmological diabetic complication	3255 (1.25)	627 (3.07)	376 (3.06)
Neurological diabetic complication	1066 (0.408)	191 (0.935)	116 (0.944)
Ischaemic stroke	8612 (3.30)	1095 (5.36)	774 (6.30)
Atrial fibrillation (AF)	7187 (2.75)	931 (4.56)	778 (6.33)
Heart failure (HF)	9107 (3.49)	1548 (7.58)	1157 (9.42)
Intracranial haemorrhage	3161 (1.19)	285 (1.40)	254 (2.07)
Ischaemic heart disease (IHD)	20 059 (7.68)	3474 (17.0)	1528 (12.4)
Osteoporosis	124 (0.047)	17 (0.083)	12 (0.098)
Hypertension	60 321 (23.1)	7564 (37.0)	4472 (36.4)
Chronic obstructive pulmonary disease	770 (0.295)	80 (0.392)	85 (0.692)

**TABLE 2:** Baseline characteristics for continuous variables in the present cohort

Characteristics	Mean ± SD		
	Total cohort (n = 261 308)	Acute myocardial infarction (n = 20 419)	Sudden cardiac death (n = 12 282)
Age	66.0 ± 11.8	71.6 ± 10.7	72.9 ± 10.6
Follow‐up duration (days)	3552 ± 1201	2949 ± 1239	2008 ± 1143
Diabetes duration (y)	4.77 ± 2.29	8.74 ± 4.12	9.95 ± 3.11
Liver function test			
Alkaline phosphatase (U/L)	79.8 ± 37.4	81.3 ± 33.7	86.3 ± 51.5
Alanine aminotransferase (U/L)	25.8 ± 24.0	22.6 ± 19.8	22.6 ± 19.3
Total protein (g/L)	74.3 ± 6.99	73.9 ± 7.24	73.1 ± 7.46
Albumin (g/L)	38.7 ± 5.39	38.0 ± 5.33	37.0 ± 5.61
Complete blood count			
Lymphocyte count (x10^9^/L)	1.88 ± 1.05	1.85 ± 0.78	1.77 ± 1.58
Neutrophil count (x10^9^/L)	5.33 ± 2.68	5.62 ± 2.76	5.70 ± 2.86
Haemoglobin count (x10^9^/L)	12.8 ± 4.29	12.4 ± 1.87	12.2 ± 1.94
Lipid profile			
High‐Density Lipoprotein‐Cholesterol (HDL‐C) (mmol/L)	1.20 ± 0.34	1.15 ± 0.33	1.17 ± 0.36
Low‐Density Lipoprotein‐Cholesterol (LDL‐C) (mmol/L)	2.92 ± 0.88	2.93 ± 0.93	2.88 ± 0.93
Total cholesterol (mmol/L)	4.84 ± 1.03	4.84 ± 1.10	4.73 ± 1.08
Triglyceride (mmol/L)	1.72 ± 1.36	1.83 ± 1.52	1.72 ± 1.38
Renal function test			
Creatinine (umol/L)	103 ± 92	128 ± 125	139 ± 152
Potassium (mmol/L)	4.22 ± 0.48	4.27 ± 0.51	4.24 ± 0.52
Sodium (mmol/L)	139 ± 3	139 ± 3	139 ± 3.54
Urea (mmol/L)	6.85 ± 4.04	8.24 ± 5.01	8.52 ± 5.61
Glycaemic control			
Fasting blood glucose (mmol/L)	7.75 ± 2.60	8.21 ± 2.00	8.12 ± 2.08
HbA1c (%)	7.44 ± 1.45	7.88 ± 1.25	7.83 ± 1.31

### 3.2 Acute myocardial infarction prediction

**FIGURE 1:**
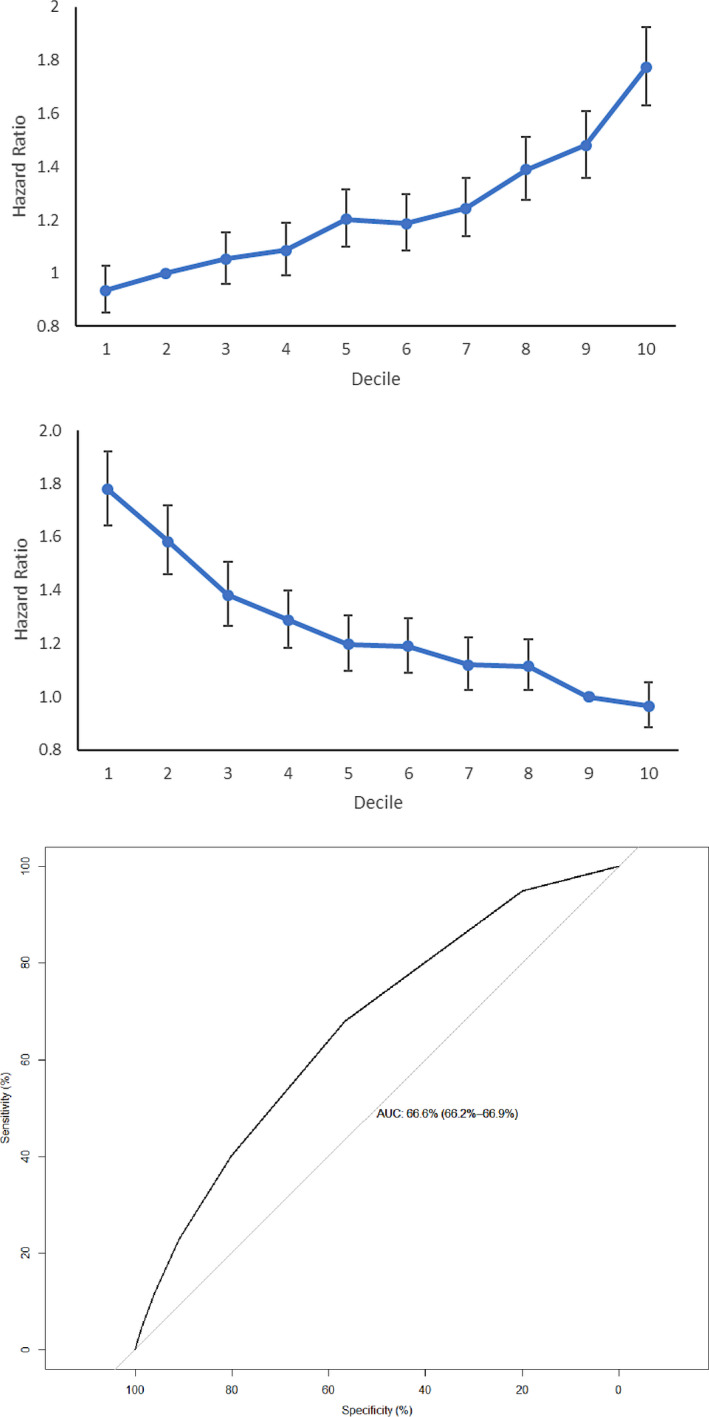
The association between mean HbA1c (top) or high‐density lipoprotein‐cholesterol (middle) and acute myocardial infarction. The receiver operator characteristic (ROC) curve for the acute myocardial infarction predictive score (bottom)

A total of 20419 patients suffered from AMI (annualized rate: 7.37%/year) with an annual frequency of 0.536 ± 8.74 episodes. The significant univariate predictors were summarized in Table S2. The following parameters were identified as significant predictors on multivariate regression (n = 34 015; Table S3): 1) age (HR=1.02, 95% CI = [1.02, 1.03], *P* < 0.0001) and male sex (HR = 1.07, 95% CI = [1.01, 1.14], *P* = 0.023); 2) baseline anaemia (HR = 1.18, 95% CI = [1.10, 1.27], *P* < 0.0001); 3) serum creatinine (HR = 1.00, 95% CI = [1.00, 1.00], *P* < 0.0001); 4) serum HDL‐C (HR = 0.802, 95% CI = [0.732, 0.878], *P* < 0.0001) and triglyceride (HR = 1.04, 95% CI = [1.03, 1.05], *P* < 0.0001); 5) comorbidities: ophthalmological diabetic complication (HR = 1.35, 95% CI = [1.22, 1.51], *P* < 0.0001), PVD (HR = 1.53, 95% CI = [1.18, 1.97], *P* = 0.001), IHD (HR = 1.59, 95% CI = [1.48, 1.71], *P* < 0.0001), hypertension (HR = 1.16, 95% CI = [1.09, 1.24], *P* < 0.001); 6) mean HbA1c (HR = 1.16, 95% CI = [1.12, 1.19], *P* < 0.0001). Details of the multivariate Cox regression are summarized in Appendix S1. Both HDL‐C and mean HbA1c showed linear relationships with AMI risk (Figure 1, top and middle panels). After adjusting for the multivariate HR of the included parameters (Table S4), a score‐based system was developed (Appendix S1). On ROC analysis, the AMI score had an AUC of 0.666 (95% CI = [0.662, 0.669]; Figure 1, bottom panel).

### 3.3 Sudden cardiac death prediction

**FIGURE 2:**
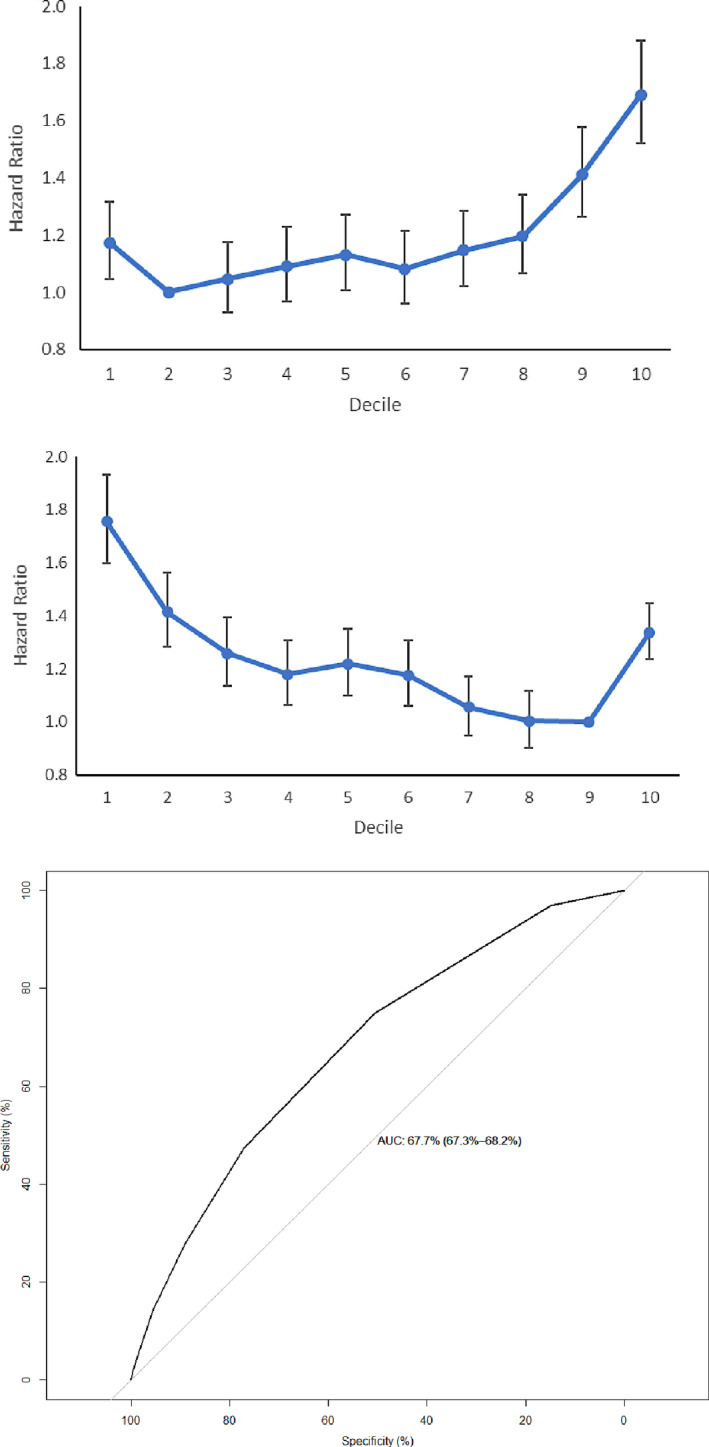
The association between mean HbA1c (top panel) and total cholesterol (middle panel) and sudden cardiac death. The receiver operator characteristic (ROC) curve for sudden cardiac death predictive score (bottom panel)

For risk stratification of SCD, 0.822% (n = 2149) patients were excluded because of AMI occurring before the SCD episode, or the SCD was associated with AMI. For this excluded subset of patients, only triglyceride levels were predictive of SCD (Appendix S1). For the remainder of the cohort, SCD occurred in 12 282 patients (annualized rate: 4.40%/year) at an annual frequency of 0.169 ± 0.569 episodes. Findings under univariate Cox regression are summarized on Table S5. Multivariate Cox regression (n = 33 423) then identified following significant predictors, which were incorporated into the predictive score (Table S6): 1) age (HR = 1.03, 95% CI = [1.02, 1.03], *P* < 0.0001) and male sex (HR = 1.34, 95% CI = [1.23, 1.45], *P* < 0.0001); 2) baseline anaemia (HR = 1.41, 95% CI = [1.29, 1.54], *P* < 0.0001); 3) serum albumin (95% CI = 0.973, 95% CI = [0.964, 0.981], *P* < 0.0001); 4) serum total cholesterol (HR = 1.04, 95% CI = [1.00, 1.08], *P* = 0.033); 5) serum creatinine (HR = 1.00, 95% CI = [1.00, 1.00], *P* < 0.0001); 5) comorbidities: ophthalmological diabetic complication (HR = 1.23, 95% CI = [1.07, 1.41], *P* = 0.004), AF (HR = 1.31, 95% CI = [1.14, 1.50], *P* < 0.0001) and HF (HR = 1.19, 95% CI = [1.06, 1.33], *P* = 0.003); 6) mean HbA1c (HR = 1.11, 95% CI = [1.07, 1.15], *P* < 0.001). Both mean HbA1c and total cholesterol demonstrated a J‐shaped relationship with non‐AMI‐related SCD (Figure 2, top and middle panels). Therefore, the cut‐offs for mean HbA1c and total cholesterol were adjusted accordingly. The multivariate HR that the marks assigned in the score are shown in Table S7. None of the variables had HRs beyond the ranges of 0.67‐1.5. Details of the score system are shown in Appendix S1, with ROC analysis showing an AUC of 0.677 (95% CI = [0.673, 0.682]) (Figure 2, bottom panel).

### 3.4 Machine learning survival analysis

A CISF model was developed to predict AMI and SCD based on the baseline clinical variables. Optimal tree number of CISF model to predict AMI was set as 700 to predict AMI, while the number was set as 600 to predict SCD, based on the fivefold cross‐validation parameter selection results as shown in Figure S1. Survival curves to predict AMI and non‐AMI‐related sudden cardiac death were generated using the CISF model (Figure S2). Variable importance values and relative importance values of variables to predict AMI and non‐AMI‐related SCD are presented in Table 3. Creatinine and age were ranked as the most important predictors of AMI, followed by baseline anaemia, mean HbA1c, triglyceride, male sex, hypertension and IHD (Figure S3, top panel). For non‐AMI‐related SCD, age and creatinine were the most important predictors, followed by baseline anaemia, mean HbA1c, HF, male sex, total cholesterol, AF, ophthalmological diabetic complication (Figure S3, bottom panel). The importance values of the different risk variables can be easily applied to construct predictive frailty scores of AMI and non‐AMI‐related SCD for clinical practice use.

**TABLE 3:** Variable importance ranking generated by CISF model

Acute myocardial infarction			Sudden cardiac death		
Variable	Importance	Relative importance	Variable	Importance	Relative importance
Creatinine (mmol/L)	0.1061	1.0000	Age, years	0.0986	1.0000
Age, y	0.0906	0.8545	Creatinine (mmol/L)	0.0923	0.9361
Baseline anaemia	0.0156	0.1469	Baseline anaemia	0.015	0.1517
Mean HbA1c (%)	0.0108	0.102	Mean HbA1c (%)	0.0126	0.1274
Triglyceride (mmol/L)	0.003	0.0284	Heart failure	0.0119	0.1208
Male sex	0.0028	0.0268	Male sex	0.0086	0.0871
Hypertension	0.002	0.0193	Total cholesterol (mmol/L)	0.0039	0.04
Ischaemic heart disease	0.0012	0.011	Atrial fibrillation	0.0024	0.0245
High‐density lipoprotein‐cholesterol (mmol/L)	0.0005	0.0045	Ophthalmological diabetic complication	0.0003	0.0032
Peripheral vascular disease	0.0001	0.0011			
Ophthalmological diabetic complication	0.0000	0.0004			

The performance of CISF model was compared with that of Random Survival Forest (RSF) model and multivariate Cox for survival analysis (Table 4) using a fivefold cross‐validation approach. CISF model significantly improves the survival performance of AMI (precision: 0.91, recall: 0.89, AUC: 0.93, C‐index: 0.91) and non‐AMI‐related SCD (precision: 0.91, recall: 0.89, AUC: 0.89, C‐index: 0.89) than RSF model and multivariate cox model.

**TABLE 4:** Comparisons between CISF, multivariate Cox and RSF model (fivefold cross‐validation)

Outcome	Acute myocardial infarction				Sudden cardiac death			
Evaluators	Precision	Recall	AUC	C‐Index	Precision	Recall	AUC	C‐Index
CISF	0.9083	0.8851	0.9270	0.9029	0.9137	0.8900	0.8912	0.8918
RSF	0.8634	0.8606	0.8506	0.8290	0.8464	0.8406	0.8691	0.8536
Multivariate Cox	0.8197	0.7568	0.7255	0.7684	0.7918	0.8276	0.7412	0.8193

## 4 DISCUSSION

There are several major findings from the present study: 1) a combination of clinical and laboratory parameters can be used to predict AMI and SCD amongst patients with type 2 diabetes mellitus; 2) J/U‐shaped relationships were not presented consistently across different cardiovascular adverse outcomes; 3) the J/U‐shaped relationships between mean HbA1c, HDL‐C, and total cholesterol and adverse cardiovascular outcomes can be incorporated into scores for clinical risk stratification; 4) CISF model identified that albumin, age, creatinine, total protein, baseline anaemia, heart failure and male gender are the most important predictors of both incident AMI and non‐AMI‐related SCD, followed by hypertension, atrial fibrillation, HDL‐C, mean fasting blood glucose for AMI while mean fasting blood glucose, hypertension and mean HbA1c for non‐AMI SCD;5) CISF significantly improves prediction performance of incident AMI and non‐AMI SCD than RSF and multivariate Cox models.

Over recent years, there is increasing reports on the J/U‐shaped relationship between both glycaemic and cholesterol indices and diabetic adverse outcomes. However, these studies mostly focused on composite outcomes, such as all‐cause mortality and major cardiovascular adverse events.^7^, ^17^, ^18^, ^19^, ^20^ Currently, there is a lack of studies looking at the relationship between HbA1c and cholesterol indices with specific cardiovascular adverse outcomes, such as AMI and SCD. In the present study, a linear relationship was observed between both mean HbA1c and HDL‐C against AMI, whilst a J‐shaped relationship was depicted for both mean HbA1c and total cholesterol against SCD. The incorporation of these biochemical variables into the risk scores yields comparable AUC to recent predictive models that involve machine‐learning techniques to account for latent interactions thus demonstrates the importance of involving biochemical indices in risk stratification.^21^

The difference that the mean HbA1c has against AMI and SCD can be explained by the different underlying pathogenic mechanisms. The linear relationship between mean HbA1c and AMI was supported by other studies with cohorts like the present study, comprised of younger patients with more diverse pre‐existing macrovascular complications, which demonstrates the importance of personalized glycaemic control.^22^, ^23^ Furthermore, coronary atherosclerosis is associated with insulin resistance, which also supports the linear relationship.^24^, ^25^ In the double‐blinded Trial Comparing Cardiovascular Safety of Insulin Degludec vs Insulin Glargine in Patients with Type 2 Diabetes at High Risk of Cardiovascular Events (DEVOTE), whilst hypoglycaemia increased the risk of cardiovascular diseases, the elevation in risk for non‐fatal AMI and unstable angina was insignificant. These findings were consistent with the present study, where low mean HbA1c is associated with an increased risk for SCD but not AMI.^26^

On a separate note, the U‐shaped relationship between mean HbA1c and SCD may be explained by the increased arrhythmic potential during both persistent hyperglycaemia and hypoglycaemia. Under chronic hyperglycaemia, persistently increased activation of calcium channels, and increased oxidative stress can induce arrhythmogenesis.^27^, ^28^, ^29^, ^30^ By contrast, hypoglycaemia is a well‐known trigger for ventricular tachyarrhythmia and is associated with delayed repolarization and altered repolarization gradients.^31^, ^32^, ^33^ During prolonged hypoglycaemia, vagal reactivation occurs and the relative bradycardia increases the risk of atrial ectopy.^34^ Severe hypoglycaemia was reported to increase the risk of arrhythmic death by 77% in the Outcome Reduction with Initial Glargine Intervention (ORIGIN) trial, which agrees with our findings.^35^ However, it should be noted that the J‐shaped relationship is mainly attributed by the lowest decile of HbA1c, suggesting that the relationship may be disrupted by extreme cases of persistent hypoglycaemia. For patients with HbA1c values within the normal range, the relationship between HbA1c and SCD was linear.

The inverse relationship between HDL‐C level and cardiovascular adverse outcomes is well established,^36^ and reinforced by recent findings of the inversed association between high lipoprotein function and atherosclerotic burden.^37^ Recent studies exploring the relationship between cholesterol indices and cardiovascular events demonstrate that the J‐shaped relationship is mainly present in LDL‐C.^20^, ^38^ The U‐shaped relationship between HDL‐C and all‐cause mortality reported may be attributed to other causes of death, such as infection and external causes, and confounded by alcoholism which raises HDL.^39^, ^40^, ^41^ These findings suggest that the J‐shaped relationship between total cholesterol and SCD may be driven by LDL‐C, given the observed linear association between HDL‐C and AMI. Given that the J‐shaped relationship between total cholesterol and SCD is mainly attributed by the highest decile for total cholesterol, there is also a possibility that the increase in SCD risk may only occur in outliers with extremely high total cholesterol.^42^ The varied pathogenesis underlying different cardiovascular adverse outcomes suggests that cause‐specific analysis on the relationship between both glycaemic and cholesterol, and cardiovascular mortality, should be performed.

The Cox proportional hazards model has been widely used as for right‐censored time‐to‐event data analysis since it is convenient for its flexibility and simplicity. However, their use is not appropriate when the proportional hazards assumption is violated. Extensions to the Cox proportional hazards model were developed but often remained dependent on restrictive functions (eg Heaviside functions) that are difficult to construct and implement. RSF models, as extensions of classification and regression trees and random forests, have been identified as alternative survival data analysis methods when the proportional hazard assumption is violated.^43^ RSF‐based models have been applied to enhance risk stratification in different clinical settings, including diabetes.^44^, ^45^, ^46^, ^47^, ^48^ However, RSF model has been criticized for the bias due to favouring covariates with many split‐points.^49^ In our study, the CISF model was used for time‐to‐event survival data analysis in predicting AMI and non‐AMI SCD,^11^, ^12^ which were shown to shown superior predictive performance compared to RSF and multivariate Cox models.

### 4.1 Limitations

Several limitations should be noted for the present study. First of all, given its observational, data‐based nature, it is susceptible to under‐coding and coding error, with an inability to establish causal relationships. In addition, the large number of patients included in the analysis drove the high statistical significance but low hazard ratio in some predictive parameters. Thus, findings of these parameters may be driven by the statistical power of the analysis and may have limited clinical significance. Furthermore, duration of diabetes was not adjusted for, given the possible competing variable of time from baseline till outcome onset. This is also to avoid interference of inaccuracy in diabetic duration because of a lack of data beyond a decade prior to baseline. Additionally, the effect of medications was not accounted for due to the potential drug‐drug interactions and effect upon the laboratory markers, which would greatly complicate the analysis. Finally, data on other cardiovascular health predictors, such as smoking status, alcohol use and family history of cardiac conditions, were unavailable due to limitations of our administrative database of not converting them into structured data for extraction.

## 5 CONCLUSION

A holistic combination of demographic, clinical and laboratory indices can be used for the risk stratification of patients with type 2 diabetes mellitus against AMI and SCD. Cause‐specific analysis should be applied to further examine the relationship between both mean HbA1c and lipid parameters against different cardiovascular adverse outcomes. The application of machine‐learning techniques can improve the sensitivity and specificity of risk prediction by identifying the latent interactions between risk variables.

## CONFLICTS OF INTEREST

None.

## AUTHORS’ CONTRIBUTIONS

SL, JZ and CG involved in data analysis, data interpretation, statistical analysis, manuscript drafting and critical revision of manuscript. WTW, ICKW, TL, WKKW and KJ involved in project planning, data acquisition, data interpretation and critical revision of manuscript. QZ and GT involved in study conception, study supervision, project planning, data interpretation, statistical analysis, manuscript drafting and critical revision of manuscript.

## Supporting information

Supinfo

Appendix

## Data Availability

The deidentified data set of this study has been deposited in Zenodo (https://zenodo.org/record/4382440) in accordance with university's policies.
