# Supplementary material for: Predictive scores for identifying patients with type 2 diabetes mellitus at risk of acute myocardial infarction and sudden cardiac death
Source: Endocrinol Diabetes Metab. 2021 Feb 19;4(3):e00240. doi: 10.1002/edm2.240 (PMC8279628; doi:10.1002/edm2.240)
Supplement: Supplementary file 1 — Supinfo [file EDM2-4-e00240-s001.docx]

**Supplementary Table 1.** The International Classification of Disease, Nineth Edition (ICD-9) codes for type 2 diabetes mellitus and the comorbidities.

| Diseases | ICD Code |
| --- | --- |
| Acute Myocardial Infarction | 410.00-410.99, 412.00-412.99 |
| Sudden Cardiac Death | 427.1, 427.4, 427.5 |
| Neurological Complications | 250.6 |
| Ophthalmological Complications | 250.5 |
| Renal Complications | 250.4 |
| Peripheral Vascular Disease | 250.7 |
| Ischemic Stroke and Transient Ischemic Attack | 433.01, 433.11, 433.21, 433.31, 433.81, 433.91, 434.01, 434.91, 436, 435 |
| Atrial Fibrillation | 427.31, 429.4 |
| Heart Failure | 428.00-428.99 |
| Ischemic Heart Disease | 410.00-411.99, 413.00-414.99 |
| Osteoporosis | 733.00 |
| Chronic Obstructive Pulmonary Disease | 490.00-496.99 |
| Hypertension | 491.00-492.99 |

**Supplementary Table 2.** Univariate predictors for acute myocardial infarction.

|  | Hazard Ratio | 95% Confidence Interval | P-Value |
| --- | --- | --- | --- |
| Age | 1.04 | [1.04, 1.05] | < 0.0001 |
| Male | 1.10 | [1.07, 1.14] | < 0.0001 |
| Diabetes Duration | 1.12 | [1.11, 1.12] | < 0.0001 |
| Mean Fasting Blood Glucose | 1.12 | [1.10, 1.13] | < 0.0001 |
| Mean HbA1c | 1.17 | [1.15, 1.18] | < 0.0001 |
| Baseline Anemia | 2.24 | [2.15, 2.33] | < 0.0001 |
| Liver Function Test | | | |
| Total Protein | 0.991 | [0.987, 0.994] | < 0.0001 |
| Albumin | 0.976 | [0.972, 0.980] | < 0.0001 |
| Renal Function Test | | | |
| Creatinine | 1.00 | [1.00, 1.00] | < 0.0001 |
| Lipid Profile | | | |
| High Density Lipoprotein Cholesterol (HDL-C) (mmol/L) | 0.587 | [0.555, 0.620] | < 0.0001 |
| Low Density Lipoprotein Cholesterol (LDL-C) (mmol/L) | 1.02 | [0.992, 1.04] | 0.185 |
| Total Cholesterol (mmol/L) | 0.999 | [0.983, 1.02] | 0.858 |
| Triglyceride (mmol/L) | 1.04 | [1.03, 1.04] | < 0.0001 |
| Comorbidity | | | |
| Renal Diabetic Complication | 2.56 | [2.36, 2.79] | < 0.0001 |
| Ophthalmological Diabetic Complication | 2.69 | [2.48, 2.91] | < 0.0001 |
| Neurological Diabetic Complication | 2.46 | [2.14, 2.84] | < 0.0001 |
| Peripheral Vascular Disease | 3.83 | [3.06, 4.78] | < 0.0001 |
| Ischemic Stroke | 1.72 | [1.61, 1.82] | < 0.0001 |
| Atrial Fibrillation | 1.75 | [1.64, 1.86] | < 0.0001 |
| Heart Failure | 2.40 | [2.28, 2.53] | < 0.0001 |
| Ischemic Heart Disease | 2.63 | [2.53, 2.72] | < 0.0001 |
| Osteoporosis | 2.03 | [1.98, 2.09] | 0.016 |
| Hypertension | 1.67 | [1.62, 1.72] | < 0.0001 |
| Chronic Obstructive Pulmonary Disease | 1.36 | [1.09, 1.69] | 0.007 |

**Supplementary Table 3.** Multivariate predictors for acute myocardial infarction.

|  | Hazard Ratio | 95% Confidence Interval | P-Value |
| --- | --- | --- | --- |
| Age | 1.02 | [1.02, 1.03] | < 0.0001 |
| Male | 1.07 | [1.01, 1.14] | 0.023 |
| Mean Fasting Blood Glucose | 0.994 | [0.976, 1.01] | 0.527 |
| Mean HbA1c | 1.16 | [1.12, 1.19] | < 0.0001 |
| Baseline Anemia | 1.18 | [1.10, 1.27] | < 0.0001 |
| Liver Function Test | | | |
| Total Protein | 1.00 | [0.996, 1.01] | 0.651 |
| Albumin | 0.998 | [0.991, 1.00] | 0.523 |
| Renal Function Test | | | |
| Creatinine | 1.00 | [1.00, 1.00] | < 0.0001 |
| Lipid Profile | | | |
| High Density Lipoprotein Cholesterol (HDL-C) | 0.802 | [0.732, 0.878] | < 0.0001 |
| Triglyceride | 1.04 | [1.03, 1.05] | < 0.0001 |
| Comorbidity | | | |
| Renal Diabetic Complication | 0.967 | [0.865, 1.08] | 0.561 |
| Neurological Diabetic Complication | 0.874 | [0.732, 1.04] | 0.132 |
| Ophthalmological Diabetic Complication | 1.35 | [1.22, 1.51] | < 0.0001 |
| Peripheral Vascular Disease | 1.53 | [1.18, 1.97] | 0.001 |
| Ischemic Stroke | 0.991 | [0.881, 1.11] | 0.883 |
| Atrial Fibrillation | 0.962 | [0.854, 1.08] | 0.518 |
| Heart Failure | 1.01 | [0.918, 1.11] | 0.862 |
| Ischemic Heart Disease | 1.59 | [1.48, 1.71] | < 0.0001 |
| Osteoporosis | 0.981 | [0.542, 1.78] | 0.950 |
| Hypertension | 1.16 | [1.09, 1.24] | < 0.0001 |
| Chronic Obstructive Pulmonary Disease | 0.848 | [0.539, 1.33] | 0.473 |

**Supplementary Table 4.** Multivariate hazard ratios of acute myocardial infarction predictive score parameters.

| Citeria | Hazard Ratio |
| --- | --- |
| Age | 1.04 |
| Male | 1.10 |
| Baseline Anemia | 1.16 |
| Creatinine | 1.00 |
| Mean HbA1c | 1.19 |
| High Density Lipoprotein Cholesterol (HDL-C) | 0.776 |
| Triglyceride | 1.05 |
| Ophthalmological Diabetic Complication | 1.35 |
| Peripheral Vascular Disease | 1.38 |
| Ischemic Heart Disease | 1.64 |
| Hypertension | 1.24 |

**Supplementary Table 5.** Univariate predictors for sudden cardiac death.

| Predictors | Hazard Ratio | 95% Confidence Interval | P-Value |
| --- | --- | --- | --- |
| Age | 1.06 | [1.05, 1.06] | < 0.0001 |
| Male | 1.23 | [1.18, 1.27] | < 0.0001 |
| Diabetes Duration | 1.21 | [1.21, 1.22] | < 0.0001 |
| Mean Fasting Blood Glucose | 1.03 | [1.01, 1.04] | < 0.001 |
| Mean HbA1c | 1.13 | [1.11, 1.15] | < 0.0001 |
| Baseline Anemia | 2.74 | [2.61, 2.87] | < 0.0001 |
| Liver Function Test | | | |
| Total Protein | 0.975 | [0.971, 0.979] | < 0.0001 |
| Albumin | 0.946 | [0.941, 0.951] | < 0.0001 |
| Lipid Profile | | | |
| High Density Lipoprotein Cholesterol | 0.818 | [0.763, 0.876] | < 0.0001 |
| Low Density Lipoprotein Cholesterol | 0.977 | [0.947, 1.01] | 0.162 |
| Total Cholesterol | 0.896 | [0.877, 0.915] | < 0.0001 |
| Triglyceride | 1.00 | [0.987, 1.02] | 0.802 |
| Renal Function Test | | | |
| Creatinine | 1.00 | [1.00, 1.00] | < 0.0001 |
| Comorbidity | | | |
| Renal Diabetic Complication | 2.94 | [2.65, 3.25] | < 0.0001 |
| Ophthalmological Diabetic Complication | 2.69 | [2.43, 2.98] | < 0.0001 |
| Neurological Diabetic Complication | 2.50 | [2.08, 3.00] | < 0.0001 |
| Peripheral Vascular Disease | 2.55 | [1.81, 3.59] | < 0.0001 |
| Ischemic Stroke | 2.03 | [1.89, 2.19] | < 0.0001 |
| Atrial Fibrillation | 2.50 | [2.32, 2.69] | < 0.0001 |
| Heart Failure | 3.07 | [2.89, 3.26] | < 0.0001 |
| Ischemic Heart Disease | 1.76 | [1.67, 1.86] | < 0.0001 |
| Osteoporosis | 2.13 | [1.21, 3.75] | 0.009 |
| Hypertension | 1.96 | [1.89, 2.03] | < 0.0001 |
| Chronic Obstructive Pulmonary Disease | 2.46 | [1.99, 3.04] | < 0.0001 |

**Supplementary Table 6.** Multivariate predictors for sudden cardiac death.

| Parameter | Hazard Ratio | 95% Confidence Interval | P-Value |
| --- | --- | --- | --- |
| Age | 1.03 | [1.02, 1.03] | < 0.0001 |
| Male | 1.34 | [1.23, 1.45] | < 0.0001 |
| Mean Fasting Blood Glucose | 0.995 | [0.972, 1.02] | 0.684 |
| Mean HbA1c | 1.11 | [1.07, 1.15] | < 0.0001 |
| Baseline Anemia | 1.41 | [1.29, 1.54] | < 0.0001 |
| Liver Function Test | | | |
| Total Protein | 1.00 | [0.994, 1.01] | 0.878 |
| Albumin | 0.973 | [0.964, 0.981] | < 0.0001 |
| Lipid Profile | | | |
| High Density Lipoprotein Cholesterol | 0.905 | [0.808, 1.01] | 0.082 |
| Total Cholesterol | 1.04 | [1.00, 1.08] | 0.033 |
| Renal Function Test | | | |
| Creatinine | 1.00 | [1.00, 1.00] | < 0.0001 |
| Comorbidity | | | |
| Renal Diabetic Complication | 1.02 | [0.890, 1.17] | 0.756 |
| Ophthalmological Diabetic Complication | 1.23 | [1.07, 1.41] | 0.004 |
| Neurological Diabetic Complication | 0.862 | [0.687, 1.08] | 0.195 |
| Peripheral Vascular Disease | 0.874 | [0.586, 1.30] | 0.510 |
| Ischemic Stroke | 1.13 | [0.981, 1.29] | 0.092 |
| Atrial Fibrillation | 1.31 | [1.14, 1.50] | < 0.0001 |
| Heart Failure | 1.19 | [1.06, 1.33] | 0.003 |
| Ischemic Heart Disease | 1.00 | [0.909, 1.11] | 0.956 |
| Osteoporosis | 1.45 | [0.755, 2.80] | 0.263 |
| Hypertension | 1.06 | [0.980, 1.16] | 0.138 |
| Chronic Obstructive Pulmonary Disease | 1.23 | [0.791, 1.91] | 0.359 |

**Supplementary Table 7.** Multivariate hazard ratios of sudden cardiac death predictive score parameters.

| Criteria | Hazard Ratio |
| --- | --- |
| Male | 1.30 |
| Age | 1.03 |
| Baseline Anemia | 1.31 |
| Albumin | 0.972 |
| Creatinine | 1.00 |
| Total Cholesterol | 1.03 |
| Mean HbA1c | 1.10 |
| Ophthalmological Diabetic Complication | 1.24 |
| Atrial Fibrillation | 1.30 |
| Heart Failure | 1.23 |

**Supplementary Figure 1.** Optimal tree number selection for CISF model (five-fold cross validation)

**
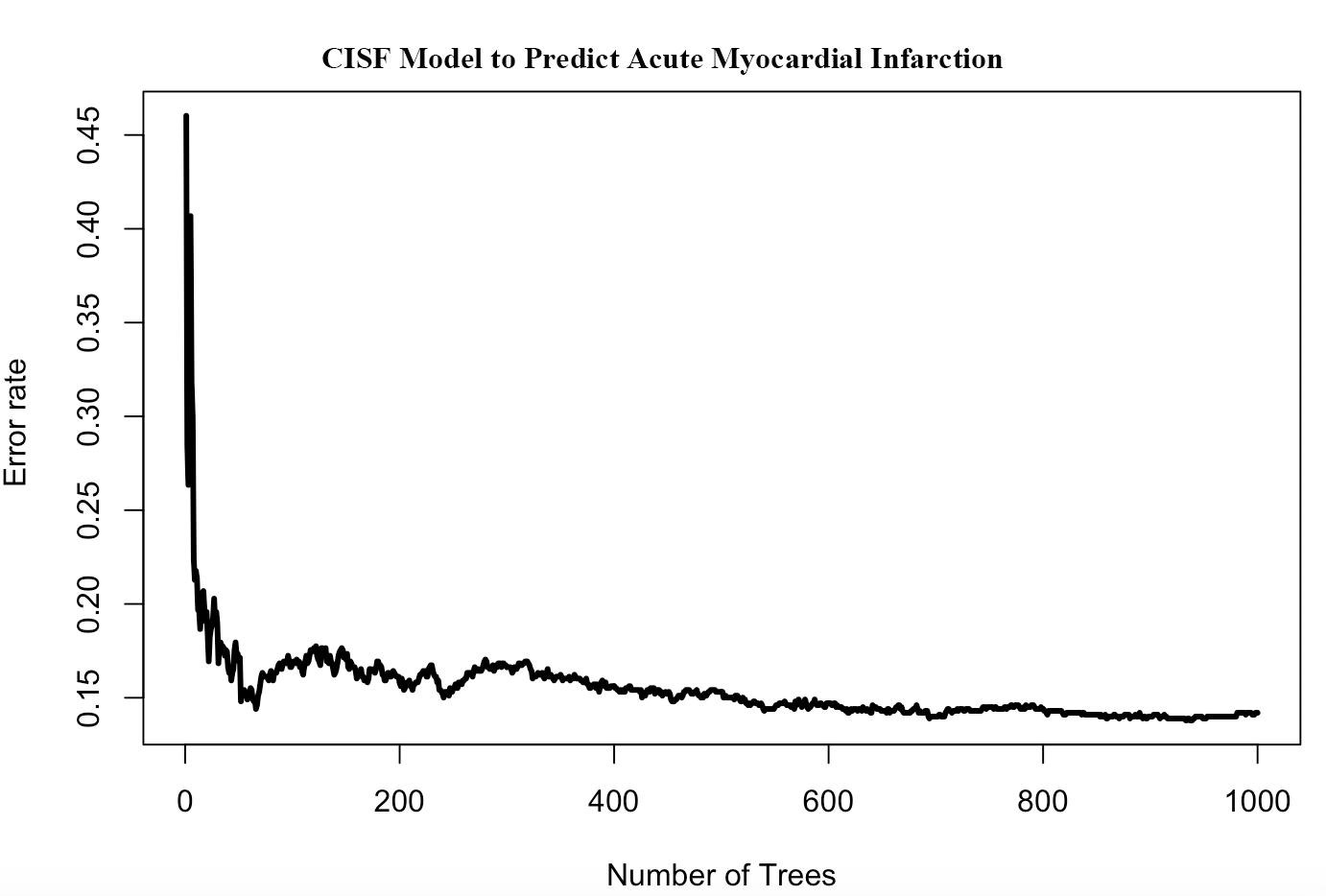
**

**
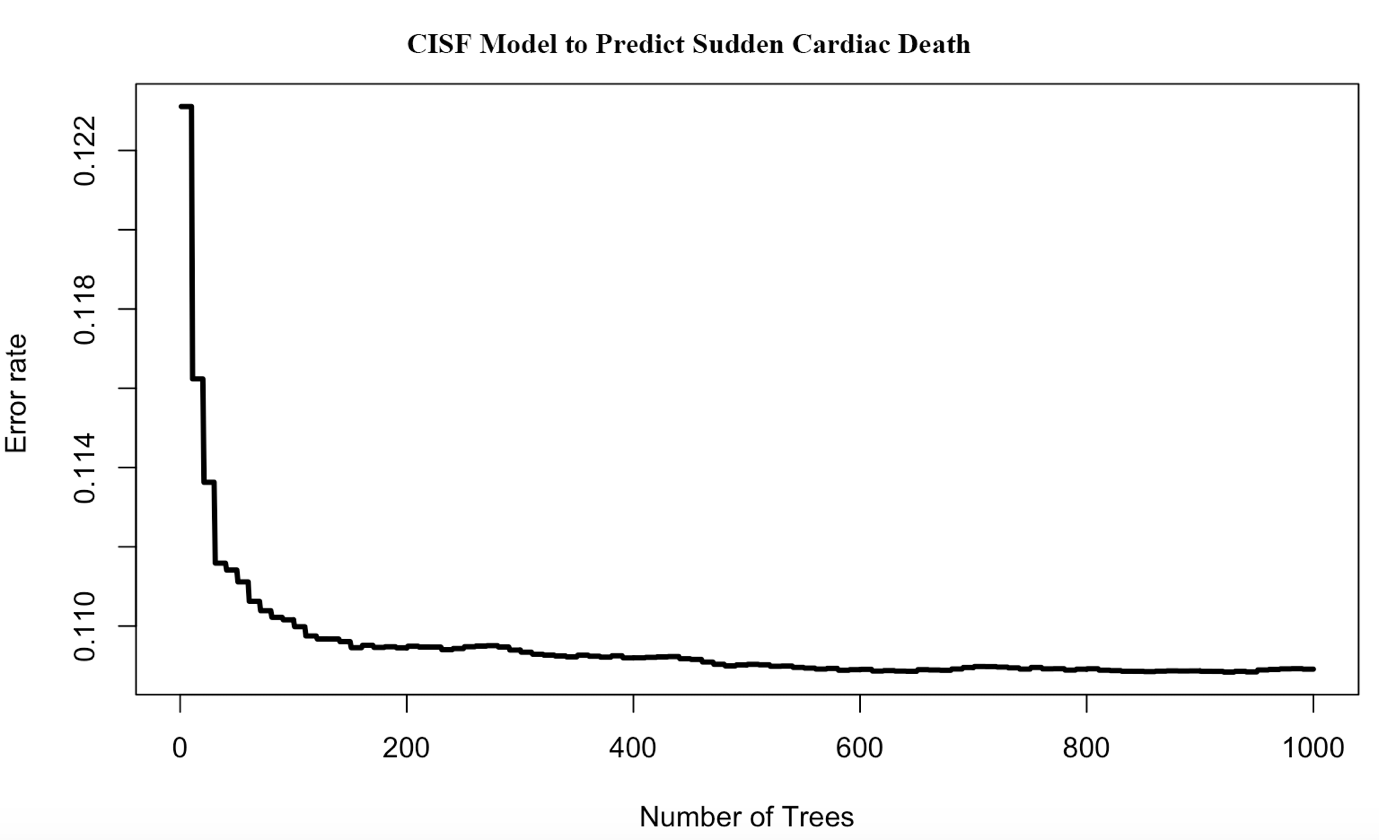
**

**Supplementary Figure 2.** Survival curves generated by CISF model for acute myocardial infarction (*top panel*) and non-AMI related SCD (*bottom panel*).

**
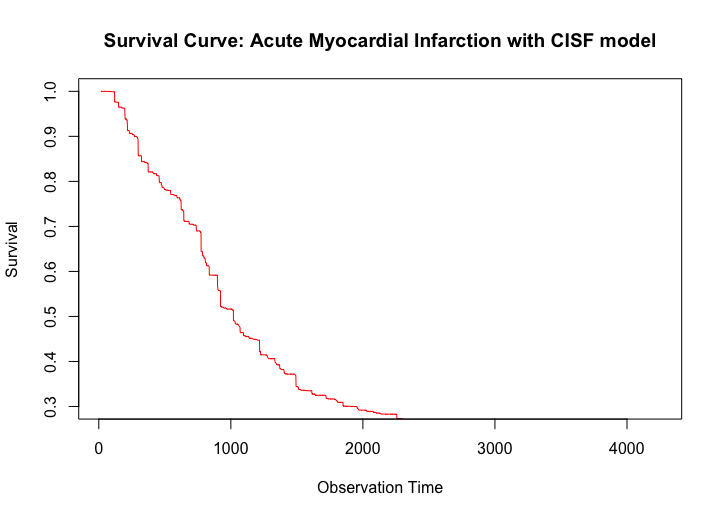
**

**
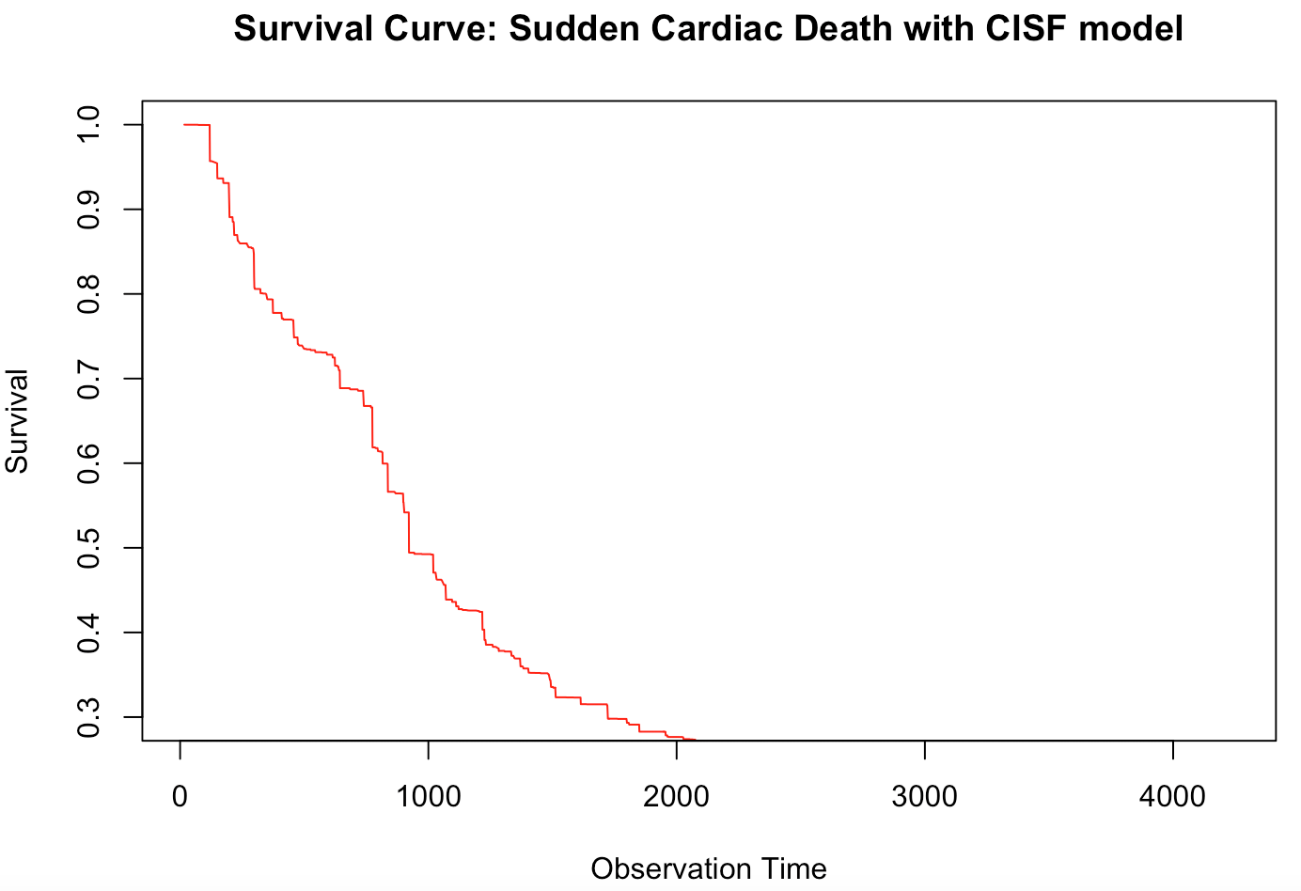
**

**Supplementary Figure 3.** Optimal tree number iteration and variable importance ranking generated by CISF model to predict acute myocardial infarction (*top panel*) or sudden cardiac death (*bottom panel*).

**
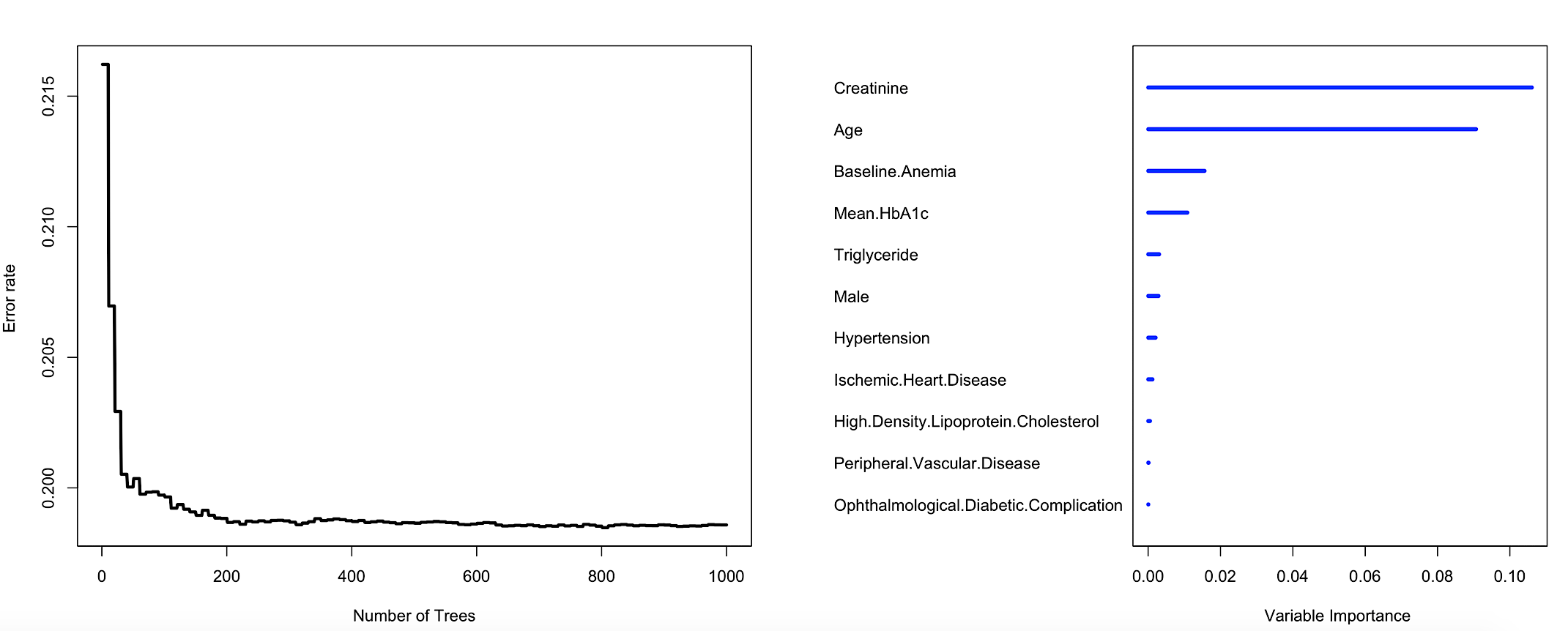
**

**
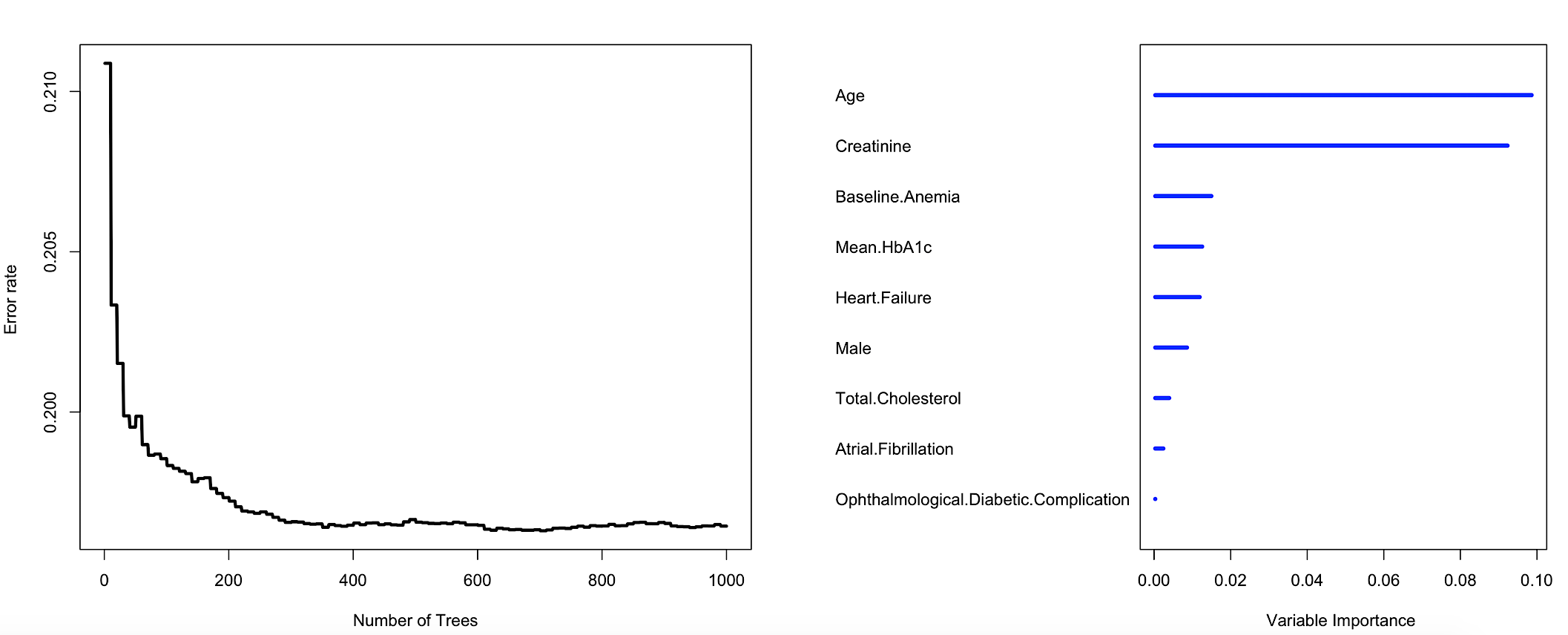
**
