# Supplementary material for: Predictive scores for identifying patients with type 2 diabetes mellitus at risk of acute myocardial infarction and sudden cardiac death
Source: Endocrinol Diabetes Metab. 2021 Feb 19;4(3):e00240. doi: 10.1002/edm2.240 (PMC8279628; doi:10.1002/edm2.240)
Supplement: Supplementary file 2 — Appendix [file EDM2-4-e00240-s002.docx]

**Supplementary Appendix**

Univariate Cox Regression.

|  | Hazard Ratio | 95% Confidence Interval | P-Value |
| --- | --- | --- | --- |
| Age | 1.00 | [0.997, 1.00] | 0.827 |
| Male | 0.953 | [0.876, 1.04] | 0.266 |
| Diabetes Duration | 1.02 | [0.999, 1.05] | 0.063 |
| Mean Fasting Blood Glucose | 1.02 | [0.996, 1.05] | 0.093 |
| Mean HbA1c | 1.04 | [0.995, 1.09] | 0.082 |
| Baseline Anemia | 1.09 | [0.977, 1.21] | 0.125 |
| Liver Function Test | | | |
| Total Protein | 0.990 | [0.981, 0.998] | 0.020 |
| Albumin | 0.991 | [0.978, 1.00] | 0.145 |
| Renal Function Test | | | |
| Creatinine | 1.00 | [1.00, 1.00] | 0.336 |
| Lipid Profile | | | |
| High Density Lipoprotein Cholesterol | 0.874 | [0.748, 1.02] | 0.089 |
| Low Density Lipoprotein Cholesterol | 0.988 | [0.927, 1.05] | 0.718 |
| Total Cholesterol | 1.02 | [0.973, 1.07] | 0.431 |
| Triglyceride | 1.07 | [1.03, 1.12] | 0.001 |
| Comorbidity | | | |
| Renal Diabetic Complication | 1.12 | [0.917, 1.37] | 0.263 |
| Ophthalmological Diabetic Complication | 1.10 | [0.904, 1.33] | 0.345 |
| Neurological Diabetic Complication | 1.24 | [0.894, 1.73] | 0.196 |
| Peripheral Vascular Disease | 1.29 | [0.779, 2.15] | 0.319 |
| Ischemic Stroke | 1.17 | [0.981, 1.40] | 0.081 |
| Atrial Fibrillation | 1.15 | [0.961, 1.39] | 0.124 |
| Heart Failure | 1.14 | [0.992, 1.31] | 0.064 |
| Ischemic Heart Disease | 1.22 | [1.09, 1.36] | < 0.001 |
| Osteoporosis | 0.849 | [0.119, 6.03] | 0.870 |
| Hypertension | 1.09 | [0.997, 1.19] | 0.059 |
| Chronic Obstructive Pulmonary Disease | 1.51 | [0.856, 2.67] | 0.155 |

Multivariate Cox Regression.

|  | Hazard Ratio | 95% Confidence Interval | P-Value |
| --- | --- | --- | --- |
| Diabetes Duration | 1.03 | [0.986, 1.09] | 0.163 |
| Mean Fasting Blood Glucose | 0.975 | [0.920, 1.03] | 0.387 |
| Mean HbA1c | 1.08 | [0.984, 1.20] | 0.101 |
| Liver Function Test | | | |
| Total Protein | 0.990 | [0.984, 1.20] | 0.097 |
| Lipid Profile | | | |
| High Density Lipoprotein Cholesterol | 0.865 | [0.645, 1.16] | 0.332 |
| Triglyceride | 1.10 | [1.02, 1.18] | 0.012 |
| Comorbidity | | | |
| Ischemic Stroke | 0.973 | [0.624, 1.52] | 0.903 |
| Heart Failure | 0.935 | [0.690, 1.27] | 0.664 |
| Ischemic Heart Disease | 1.17 | [0.922, 1.48] | 0.199 |
| Hypertension | 0.977 | [0.790, 1.21] | 0.828 |

Acute myocardial infarction prediction score.

| Criteria | Cut-off | Score |
| --- | --- | --- |
| Age (years) | >70 | 1 |
| Sex | Male | 1 |
| Baseline Anaemia | Present | 1 |
| Creatinine (mmol/L) | >64.00 | 1 |
| High Density Lipoprotein Cholesterol (mmol/L) | <1.07 | 1 |
| Mean HbA1c (%) | > 8.51 | 1 |
| Triglyceride (mmol/L) | >1.44 | 1 |
| Ophthalmological Diabetic Complication | Present | 1 |
| Peripheral Vascular Disease | Present | 1 |
| Ischemic Heart Disease | Present | 2 |
| Hypertension | Present | 1 |

Sudden cardiac death predictive score.

| Criteria | Cut-off | Score |
| --- | --- | --- |
| Age | >67 | 1 |
| Sex | Male | 1 |
| Baseline Anaemia | Present | 1 |
| Total Cholesterol (mmol/L) | <5.00 or >6.11 | 1 |
| Creatinine (mmol/L) | >93.6 | 1 |
| Mean HbA1c (%) | <6.33 or > 7.79 | 1 |
| Ophthalmological Diabetic Complication | Present | 1 |
| Atrial Fibrillation | Present | 1 |
| Heart Failure | Present | 1 |
